# Validation of Deep Learning–Based Automatic Retinal Layer Segmentation Algorithms for Age-Related Macular Degeneration with 2 Spectral-Domain OCT Devices

**DOI:** 10.1016/j.xops.2024.100670

**Published:** 2024-12-04

**Authors:** Souvick Mukherjee, Tharindu De Silva, Cameron Duic, Gopal Jayakar, Tiarnan D.L. Keenan, Alisa T. Thavikulwat, Emily Chew, Catherine Cukras

**Affiliations:** 1 Clinical Trials Branch, Division of Epidemiology & Clinical Applications, National Eye Institute, National Institutes of Health, Bethesda, Maryland; 2 Johnson and Johnson, New Brunswick, New Jersey; 3 Roche Pharmaceuticals, Basel, Switzerland

**Keywords:** Age-related macular degeneration, Artificial intelligence, Generalizability, Inter device segmentation, OCT segmentation

## Abstract

**Purpose:**

Segmentations of retinal layers in spectral-domain OCT (SD-OCT) images serve as a crucial tool for identifying and analyzing the progression of various retinal diseases, encompassing a broad spectrum of abnormalities associated with age-related macular degeneration (AMD). The training of deep learning algorithms necessitates well-defined ground truth labels, validated by experts, to delineate boundaries accurately. However, this resource-intensive process has constrained the widespread application of such algorithms across diverse OCT devices. This work validates deep learning image segmentation models across multiple OCT devices by testing robustness in generating clinically relevant metrics.

**Design:**

Prospective comparative study.

**Participants:**

Adults >50 years of age with no AMD to advanced AMD, as defined in the Age-Related Eye Disease Study, in ≥1 eye, were enrolled. Four hundred two SD-OCT scans were used in this study.

**Methods:**

We evaluate 2 separate state-of-the-art segmentation algorithms through a training process using images obtained from 1 OCT device (Heidelberg-Spectralis) and subsequent testing using images acquired from 2 OCT devices (Heidelberg-Spectralis and Zeiss-Cirrus). This assessment is performed on a dataset that encompasses a range of retinal pathologies, spanning from disease-free conditions to severe forms of AMD, with a focus on evaluating the device independence of the algorithms.

**Main Outcome Measures:**

Performance metrics (including mean squared error, mean absolute error [MAE], and Dice coefficients) for the segmentations of the internal limiting membrane (ILM), retinal pigment epithelium (RPE), and RPE to Bruch’s membrane region, along with en face thickness maps, volumetric estimations (in mm^3^). Violin plots and Bland–Altman plots comparing predictions against ground truth are also presented.

**Results:**

The UNet and DeepLabv3, trained on Spectralis B-scans, demonstrate clinically useful outcomes when applied to Cirrus test B-scans. Review of the Cirrus test data by 2 independent annotators revealed that the aggregated MAE in pixels for ILM was 1.82 ± 0.24 (equivalent to 7.0 ± 0.9 μm) and for RPE was 2.46 ± 0.66 (9.5 ± 2.6 μm). Additionally, the Dice similarity coefficient for the RPE drusen complex region, comparing predictions to ground truth, reached 0.87 ± 0.01.

**Conclusions:**

In the pursuit of task-specific goals such as retinal layer segmentation, a segmentation network has the capacity to acquire domain-independent features from a large training dataset. This enables the utilization of the network to execute tasks in domains where ground truth is hard to generate.

**Financial Disclosure(s):**

Proprietary or commercial disclosure may be found in the Footnotes and Disclosures at the end of this article.

Spectral-domain OCT (SD-OCT) images are commonly used for the diagnosis and monitoring of outer retinal diseases, such as age-related macular degeneration (AMD).^1^, ^2^, ^3^ The automatic segmentation of retinal layers in SD-OCT images could help in identifying abnormalities induced by diseases in large-scale clinical studies. With the progress of machine learning techniques, numerous deep learning methods have been developed for retinal layer segmentation.^4^, ^5^, ^6^, ^7^, ^8^ Deep learning algorithms are most often considered device-dependent with respect to the differences in image quality and preprocessing/postprocessing steps deployed by device manufacturers,^9^, ^10^, ^11^ as they are trained and used on a single device's output. Hence, it cannot be assumed that the algorithms generalize across devices, and they need to be extensively validated to ensure their ability to precisely segment changes in the presence of disease and robustly compute disease-related metrics. This validation is essential for effective translation and widespread adoption of these algorithms.

A common challenge for most deep learning algorithms is that large numbers of labeled OCT images are required for training and annotations are time-consuming and require involvement of expert physicians or graders. Moreover, the image acquisition settings of the training dataset often differ from the test dataset, where device manufacturer, imaging parameters, and preprocessing/postprocessing methods are often different. For example, the Zeiss Cirrus and Heidelberg Spectralis are 2 frequently used device manufacturers in retinal SD-OCT imaging, where differences in the image appearance/resolution are often visibly apparent. Even with the same instrument manufacturer, images from different devices can vary over time due to the application of advanced acquisition and processing methods. This challenges the development of deep learning algorithms where it is often not possible to acquire training datasets ahead of time to replicate the test setting. Even when the test setting variables are known, generating ground truth data for retraining algorithms can be cumbersome due to time and cost limitations.

Recent deep learning studies^12^ have developed algorithms to segment retinal layers from unseen B-scans captured by devices not used during model training. However, this study^12^ trained the model using only 110 labeled Spectralis Heidelberg OCT B-scans and tested it on just 60 OCT B-scans from the Topcon 1000 device. The impact of using a model trained with only a very limited training set when tested on a large external clinical trial dataset remains unclear.

In this work, we aim to validate retinal OCT segmentation algorithms across different device manufactures in their ability to generalize the successful segmentation of retinal layers in the presence of AMD disease-related changes. When dealing with several imaging devices exhibiting varying image quality, algorithm design considerations arise regarding whether to employ domain-adaptation techniques to utilize distinct models tailored to specific test device manufacturers/settings or create a resilient single model capable of generalizing across diverse manufacturers/settings. In this work, we utilize 2 widely used SD-OCT image manufacturers to test whether algorithms developed using images acquired from 1 device (Heidelberg Spectralis) could translate to another device (Zeiss Cirrus) without having to fine-tune the image quality, intensity, or signal-to-noise parameters of the latter. We evaluate 2 different state-of-the-art image segmentation networks (i.e., U-Net^13^ and DeepLabV3^14^) that have shown promise in retinal layer segmentation of patients with AMD. We use images from a Heidelberg Spectralis HRA + OCT system to train, develop, and validate the segmentation models. We then use the developed models to test and validate images from the hold-out Heidelberg test set and images acquired from the Zeiss Cirrus SD-OCT device. The 2 segmentation models were validated first using standard distance-based metrics to test the agreement with the human-defined segmentations. We then compared automatic volumetric measurements computed from different models to assess the agreement across different models and device manufacturers with manual measurements.

## Methods

### Dataset Description

The dataset was obtained from individuals participating in a prospective longitudinal study on AMD at the National Eye Institute, National Institutes of Health (NCT01352975, www.clinicaltrials.gov).^15^ Adults >50 years of age with no AMD (AMD severity score 0) to advanced AMD (presence of central geographic atrophy [AMD severity score 9–10] and/or choroidal neovascularization [AMD severity score 11]), as defined in the Age-Related Eye Disease Study,^16^ in ≥1 eye, were enrolled. The study was approved by the Institutional Review Board of the National Institutes of Health and followed the tenets of the Declaration of Helsinki. All participants provided informed written consent, after the nature and consequences of the study were explained.

Multimodal images including SD-OCT images using the Heidelberg Spectralis HRA + OCT system (Heidelberg Engineering Inc) and Cirrus OCT images (Carl Zeiss AG) were acquired, along with color fundus photographs using the TRC-50DX retinal camera (Topcon Medical Systems), at annual study visits. The color fundus photographs were graded by the Wisconsin Reading Center and assigned AMD severity grades from 0 to 11.^17^

The dataset contains images that span the full spectrum of AMD severity, graded from 0 to 11. This variability in pathology presents a challenge for any learning algorithm. The bins were created according to their AMD severity scores, as detailed in Table 1. In this table, we present the number of unique eyes (n eyes), patient age, the number of OCT visits recorded, and the percentage of pseudophakic visits relative to the total OCT visits at the time of image acquisition. Of the 402 analyzed visits, demographic information was available for 386.

**Table 1:** Patient Demographics

AMD Severity Score	n Eyes	Age (Yrs)	Number of OCT Volumes	Pseudophakic (%)
0	34	73.7 + 10.6	57	40.4
1	17	67.7 + 6	24	8.3
2	27	69.1 + 14.6	34	20.6
3	16	68.4 + 8.3	17	23.5
4	33	69.6 + 9.3	41	24.4
5	20	68.6 + 9.3	24	25.0
6	39	71.3 + 10.2	45	31.1
7	55	72.8 + 8.3	59	44.1
8	22	65.8 + 8.2	31	25.8
9	12	72.0 + 8.5	12	33.3
10	8	65.1 + 12.4	8	12.5
11	28	75.7 + 8.6	34	47.1

AMD = age-related macular degeneration.

The Spectralis SD-OCT images were volumetric macular scans of 496 × 768 × 121 pixels, spanning 30° horizontally and 25° vertically, corresponding to 1.9 × 9 × 7 mm, with a spacing of 3.87 × 11.7 × 58.7 μm between the frames of the 3 spatial axes. Infrared and OCT 30° automatic real-time tracking scans from Heidelberg Eye Explorer^18^ version 1.10.0.0 were exported. Three distinct surfaces segmented (internal limiting membrane [ILM], retinal pigment epithelium [RPE], and Bruch’s membrane [BM] layers) using Heidelberg proprietary software in each of the 496 × 768 B-scans were manually corrected, where necessary, by graders at the Wisconsin Reading Center.

The Cirrus SD-OCT images were volumetric macular scans of 512 × 512 × 128 pixels, corresponding to 2 × 6 × 6 mm, with a spacing of 3.91 × 11.7 × 47.2 μm between the frames of the 3 spatial axes. These SD-OCT images were exported from the Cirrus device. We performed interpolation on Spectralis and Cirrus OCT images to align the 2 image sets, ensuring consistency in the representation of physical macular regions.

A total of 402 3-dimensional (3D) OCT images were used for the experiments in this study. We used 201 interpolated Spectralis OCT images, selected randomly at the participant level, to train the models, and the remaining 201 hold-out interpolated Spectralis and 201 Cirrus OCT images to test performance. The number of samples (201 volumes) correspond to 201 × 128 = 25 728 B scans.

Notably, increasing the number of training samples beyond 201 volumes (equating to 25 728 B-scans) did not result in a decrease in training loss, indicating that the models had encountered sufficient variability in the training images, leading to stabilization of the loss. The networks underwent training for 5 epochs with a learning rate of 1e^-05^, utilizing the Adam optimizer.

### Manual Ground Truth Annotation

Both segmentation networks were trained using Spectralis B-scans and corresponding ground truth segmentation labels provided by a reading center, delineating the boundaries of the ILM, RPE, and the RPE drusen complex (RPEDC) region area.

Performance of the segmentation networks on test images from Spectralis was compared to the segmentations of ILM, inner RPE, and BM layers from the reading center. For Cirrus, segmentations of the ILM, inner RPE, and outer RPE layers were obtained from 28 volumes (28 OCT images × 128 B-scans per image = 3584 B-scans) using OCT Explorer software^18^, ^19^, ^20^, ^21^ and reviewed and corrected by 2 independent annotators (G.J. and C.D.). The BM layer was manually recreated by adjusting the layer corresponding to the outer RPE layer. Performance of the segmentation networks on Cirrus test images was compared to ground truth segmentations of ILM, inner RPE, and BM layers created by the 2 annotators.

**Figure 1:**
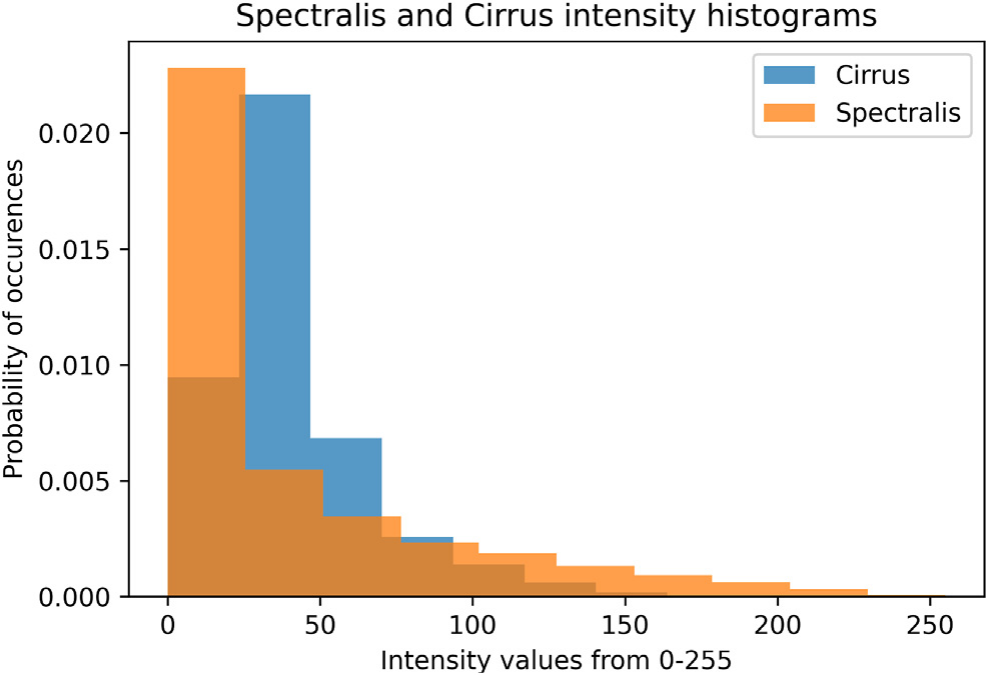
Histogram comparison of image intensities between Spectralis and Cirrus for the 28 images with Cirrus layer annotations. The probability of pixel occurrence is plotted against raw intensity values ranging from 0 to 255.

For all 28 annotated Cirrus test volumes, we performed a pairwise comparison of the intensities between the corresponding Spectralis and Cirrus OCT volumes (histogram shown in Fig 1). The R value was 0.29, indicating a low correlation between the intensities of the different imaging devices. A 2-tailed paired *t* test revealed a *P* value of <0.001, showing significant differences in the image intensities between the 2 devices.

### U-Net Segmentation Network

U-Net has proven to be a powerful tool for retinal layer segmentation, particularly in medical image analysis. Its architecture, characterized by a contracting and expansive path, allows for effective feature extraction and precise localization of retinal layers. The U-Net's ability to capture hierarchical contextual information has made it a popular choice in accurately delineating retinal structures, contributing to advancements in the diagnosis and treatment of various ocular conditions. The U-Net^13^ deep learning segmentation network trained on images from Spectralis was used to perform retinal layer segmentation in this work.

### DeepLabV3 Segmentation Network

The DeepLabV3 model excels in semantic segmentation tasks, including the intricate task of delineating retinal layers with high precision. Its utilization of dilated convolutions helps capture contextual information at multiple scales (at local and global scales), enabling accurate identification and segmentation of retinal structures in medical imaging applications. In this study, we employ the DeepLabV3^14^ segmentation network trained on Spectralis images to delineate retinal layers from OCT scans. This choice of networks is motivated by the recognition that retinal OCT B-scans, whether affected by disease or not, exhibit both local and global features influencing the retinal layers, particularly in complex cases with advanced pathology.

**Figure 2:**
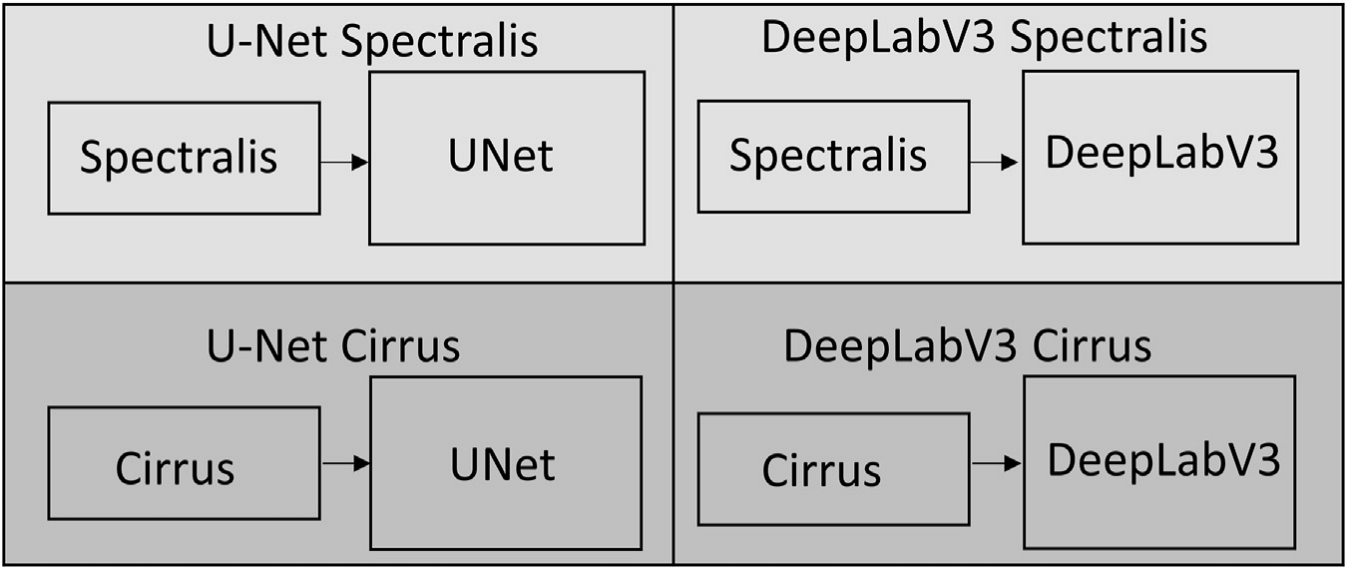
Block diagram depicting the 4 different model settings during testing phase.

Both the networks (U-Net and DeepLabV3) were trained only using Spectralis images. Among the 2 different segmentation networks and data from 2 device manufactures, we perform validation in 4 different settings: (1) U-Net model tested on hold-out Spectralis data: U-Net (Spectralis), (2) DeepLabV3 model tested on hold-out Spectralis data: DeepLabV3 (Spectralis), (3) U-Net model tested on Cirrus data: U-Net (Cirrus), and (4) DeepLabV3 model tested on Cirrus data: DeepLabV3 (Cirrus). Figure 2 demonstrates these 4 different settings.

### Automatic SD-OCT Metrics and Validation

The segmentation networks produced labels spanning multiple pixels, and to determine the boundaries of the ILM and RPE layers, we extracted medians along the columns of the B-scans. For the BM layer within the RPEDC area, we extracted a pixel near the RPEDC boundary edge, just above the choroid, corresponding to the BM layer. Consistently, we extracted the 95th percentile pixel from the RPEDC column to define the BM, avoiding potential noise associated with the last pixel or the 100th percentile pixel. This choice prevents the extraction of a noisy BM layer, where pixels might belong to the choroid rather than the RPEDC boundary, particularly in cases of advanced disease or geographic atrophy with minor segmentation errors. The extraction of ILM and RPE was performed at the 50th percentile of the ILM and RPE column labels, respectively, while the BM was extracted at the 95th percentile of the RPEDC column labels, as the BM occurs at the end of the RPEDC region. It is important to note that the BM at the 95th percentile of the RPEDC area was solely extracted for visual representation in Figure 2 and was not employed for any other qualitative or quantitative analysis.

All 4 networks were trained using available annotations from Spectralis B-scans created by the reading center. During validation, DeepLabV3 *(Spectralis)* was tested using Spectralis images, and *DeepLabV3 (Cirrus)* was tested using Cirrus images. *U-Net (Spectralis)* was tested using Spectralis images, and *U-Net (Cirrus)* model was tested using Cirrus images. For the 2 models that were tested using Cirrus images, we compared the model prediction results against 2 sets of ground truths that were annotated by 2 independent trained annotators (annotator 1 [G.J.] and annotator 2 [C.D.]). We report the aggregate annotation results. We report the mean absolute error (MAE) and mean squared error (MSE) to compare model predictions with the ground truth. If a layer is interrupted or missing in either the prediction or the ground truth, those pixels are excluded from the MAE and MSE calculations. This approach is necessary because both predictions and ground truth data are required for accurate metric computation.

Evaluation of model performances was also done by comparing the neurosensory retina (NSR) volumes (i.e., the volume bounded by the ILM and RPE layers) and RPEDC volumes (i.e., the volume bounded by the RPE and BM layers) from the model predictions versus ground truth grading of the Heidelberg images obtained from the Wisconsin reading center, as this metric should be comparable across devices. We also report the distance metrics between the predicted ILM and RPE layers compared to the manually created ground truth and report the Dice similarity coefficients for the segmented RPEDC regions.

We present the findings by categorizing the eyes based on their Age-related Macular Degeneration Severity Scores (AMDSCs), following the Age-Related Eye Disease Study 2 report. In Table 2, Table 3, Table 4, Group 1 includes eyes with AMDSCs 0 and 1 (i.e., without AMD); Group 2 encompasses eyes with AMDSCs 2, 3, 4, and 5; and Group 3 consists of eyes with AMDSCs 6, 7, and 8. Specifically, in the test set, Group 1 denotes eyes without AMD (41 Spectralis and 3 Cirrus), Group 2 represents eyes with low AMD (68 Spectralis and 3 Cirrus), and Group 3 characterizes eyes with intermediate AMD (iAMD) accompanied by medium to large drusen (57 Spectralis and 22 Cirrus).

**Table 2:** Performance Comparison for U-Net and DeepLab Models Evaluated in Cirrus and Spectralis Images

AMDSC Groups	ILM (in Pixels)		RPE (in Pixels)	
	MSE	MAE	MSE	MAE
U-Net (Spectralis)				
Group 1	0.83 ± 0.32	0.54 ± 0.20	1.09 ± 0.62	0.87 ± 0.64
Group 2	0.85 ± 0.28	0.59 ± 0.20	0.84 ± 0.27	0.58 ± 0.26
Group 3	1.27 ± 1.86	0.73 ± 1.03	2.13 ± 2.38	1.72 ± 2.26
DeepLab (Spectralis)				
Group 1	0.80 ± 0.33	0.51 ± 0.19	1.06 ± 0.50	0.84 ± 0.52
Group 2	0.80 ± 0.17	0.54 ± 0.17	0.85 ± 0.21	0.59 ± 0.19
Group 3	1.06 ± 1.41	0.61 ± 0.60	1.79 ± 1.16	1.31 ± 0.87
U-Net (Cirrus)				
Group 1	2.09 ± 0.40	1.85 ± 0.37	2.45 ± 0.69	2.58 ± 1.12
Group 2	2.01 ± 0.22	1.82 ± 0.24	2.88 ± 0.47	3.10 ± 1.13
Group 3	2.33 ± 0.93	1.98 ± 0.40	2.96 ± 1.13	3.56 ± 2.06
DeepLab (Cirrus)				
Group 1	2.39 ± 1.75	2.45 ± 2.32	2.70 ± 0.61	2.46 ± 0.66
Group 2	2.05 ± 0.27	1.86 ± 0.31	3.00 ± 0.41	2.82 ± 0.45
Group 3	2.40 ± 1.15	2.18 ± 1.32	3.42 ± 0.87	2.99 ± 0.76

AMDSC = Age-related Macular Degeneration Severity Score; ILM = internal limiting membrane; MAE = mean absolute error; MSE = mean squared error; RPE = retinal pigment epithelium.

Mean square error and MAE for ILM and RPE layers for 3 different groups according to AMDSCs; 1 pixel ∼ 3.9 μm.

**Table 3:** Intergrader Variability Measured as RMSE between Annotators for Retinal Layers of Cirrus SD-OCT Images

AMDSC Groups	ILM (in Pixels)	RPE (in Pixels)
Group 1	0.23	1.26
Group 2	0.15	1.24
Group 3	0.45	4.44

AMDSC = Age-related Macular Degeneration Severity Score; ILM = internal limiting membrane; RMSE = root mean squared error; RPE = retinal pigment epithelium; SD-OCT = spectral-domain OCT.

1 pixel ∼ 3.9 μm.

### Comparison with CycleGAN-Based Domain Adaptation

The CycleGAN network^22^ was developed to enable domain adaptation by learning the distribution of the target domain, facilitating domain shifts without requiring aligned or registered images from both source and target domains. Previously, CycleGAN has demonstrated improvements in segmenting fluid deposits and photoreceptor layers in SD-OCT images by reducing interdevice variability across images from different vendors.^10^ More recently, generative adversarial networks have been explored to segment retinal layers and intraretinal fluid,^12^ yet the limited dataset size—particularly for diseased eyes—restricts the robustness of the segmentation results. Specifically, the small number of B-scans used in both training and evaluation presents a challenge, as diseased eyes, with their complex and abnormal anatomical features, are particularly difficult to segment. The referenced study reported a moderate Dice score of 58% for intraretinal fluid segmentation in such cases, indicating challenges in handling pathological cases.

Our proposed comparison network combines a CycleGAN for domain adaptation followed by either U-Net or DeepLab as the segmentation model. This domain adaptation and segmentation framework operates as a single, end-to-end network.

In our approach, while we do not have pixel-to-pixel alignment between the source (Cirrus) and target (Spectralis) domains, we use macula image pairs representing identical physical regions, ensuring that paired images cover the same spatial area within the macula. We implemented 2 models for comparative evaluation: CycleGAN + U-Net (Cirrus) and CycleGAN + DeepLab (Cirrus), where the CycleGAN network precedes U-Net and DeepLab in an end-to-end configuration. Both models were trained on Spectralis images and tested on domain-adapted Cirrus images (adapted to Spectralis). The train and test sets were as described previously in the section “Dataset Description.” For domain-adapted Cirrus test images, we evaluated model predictions against ground truths annotated independently by 2 trained annotators to ensure comprehensive performance assessment. We report the aggregate results in Tables 5 and 6.

## Results

### Evaluation of Segmentation Models—Distance Error–Based Validation

Table 2 compares the predictive performance of the U-Net and DeepLabV3 models across Spectralis and Cirrus images, identified as U-Net (Spectralis), DeepLabV3 (Spectralis), U-Net (Cirrus), and DeepLabV3 (Cirrus). The assessment focused on the ILM and RPE layers, employing 2 distance-based metrics, namely MSE and MAE. These metrics quantified the discrepancies between model predictions and human annotations, which were provided in pixel-wise layer annotations.

In comparison to the human variability in annotations, both U-Net and DeepLabV3 models demonstrated successful performance in segmenting the retinal layers-of-interest from the B-scans to an efficient degree. Internal limiting membrane layer segmentation exhibited low errors for both models as well as the 2 OCT imaging devices. Acknowledging the challenges in robustly segmenting RPE, it can be noted that DeepLabV3 model performed slightly better in Spectralis test data. For Cirrus test data, both models performed comparably and worse than the Spectralis test data.

Table 7 presents the results of a pairwise 2-tailed *t* test comparing the MAE for model predictions between Cirrus and Spectralis images. The significance threshold was set at *P* = 0.005. The MAE for the ILM and RPE layers showed no significant differences between Cirrus and Spectralis when segmented using UNet and DeepLabV3. The table displays the *P* values obtained from the *t* test.

Table 3 illustrates the intergrader variability for Cirrus layer annotations (for the 28 annotated Cirrus images), quantified as the root MSE between human annotators. Given the complexities involved in reliably detecting changes to the retinal layers, understanding the measured human variability is pivotal for contextualizing algorithm performance. Retinal pigment epithelium exhibited considerably higher variability compared to ILM. Moreover, groups with advanced AMD, particularly Group 3, demonstrated the poorest repeatability among human annotations.

**Table 4:** Volumetric Dice Similarity Coefficients for RPEDC Region

AMDSC Groups	U-Net (Spectralis) Ground Truth: Wisconsin Reading Center	DeepLabV3 (Spectralis) Ground Truth: Wisconsin Reading Center	UNet (Cirrus) Ground Truth: Annotator 1 & 2 (Aggregated)	DeepLabV3 (Cirrus) Ground Truth: Annotator 1 and 2 (Aggregated)
Group 1	0.83 ± 0.03	0.84 ± 0.02	0.86 ± 0.01	0.87 ± 0.01
Group 2	0.84 ± 0.01	0.87 ± 0.01	0.85 ± 0.01	0.86 ± 0.01
Group 3	0.76 ± 0.04	0.79 ± 0.04	0.78 ± 0.04	0.78 ± 0.03

AMDSC = Age-related Macular Degeneration Severity Score; RPEDC = retinal pigment epithelium drusen complex.

The source of the ground truth for the specific test domain device is mentioned in the headers.

The overall interannotator variability, or the difference between annotators, was determined to be 0.76 ± 2.4 pixels (1 pixel ∼ 3.9 μm for Cirrus).

**Figure 3:**
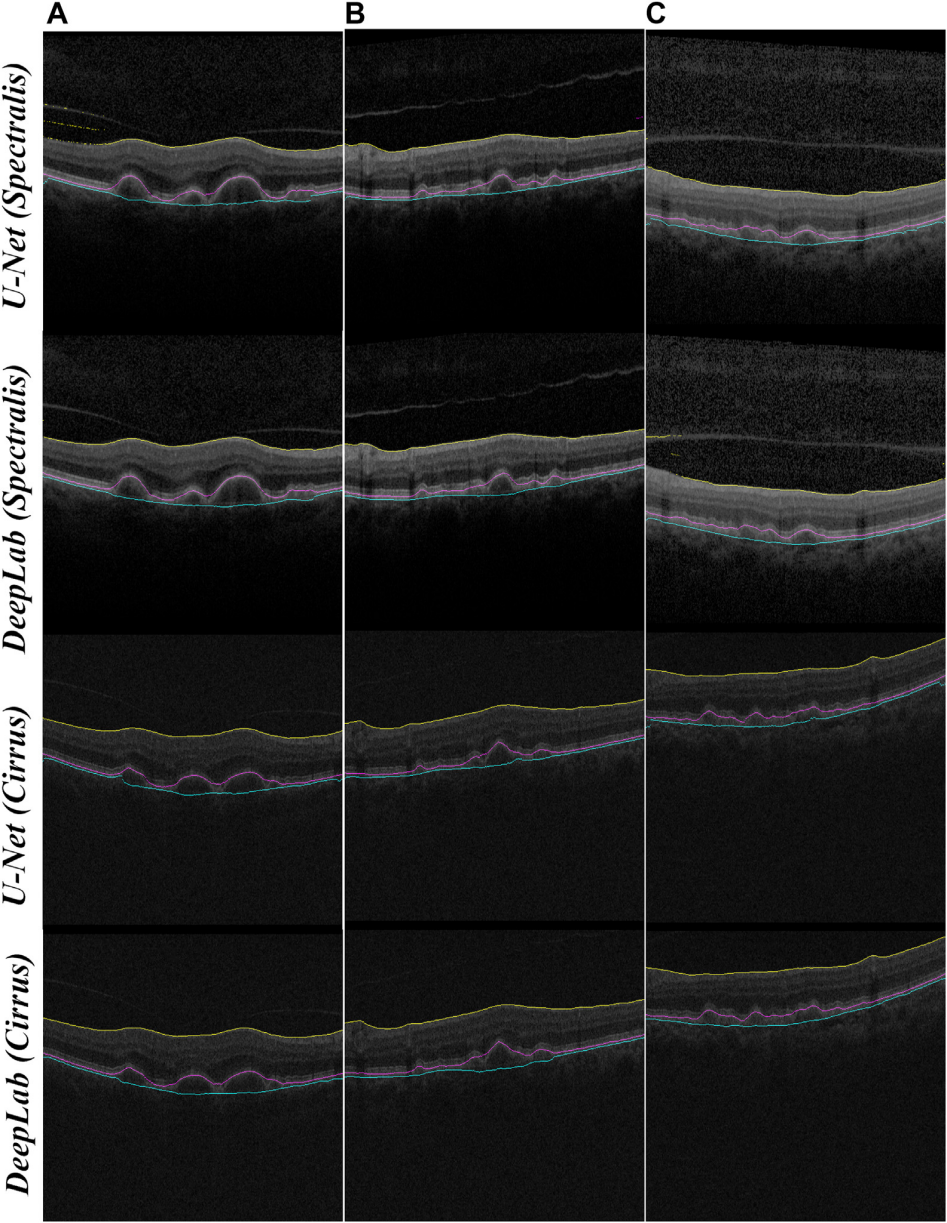
Columns (**A**), (**B**), and (**C**) show 3 B-scans from 3 patients with varying drusen volumes. All models were trained using original Spectralis B-scans. Rows (1), (2), (3), and (4) show the outputs of the models when U-Net is tested using the Spectralis images, DeepLabV3 is tested using Spectralis images, UNet is tested using Cirrus images, and DeepLabV3 is tested using Cirrus images, respectively. The output prediction is overlapped with the input test B-scans from respective Spectralis or Cirrus devices. The ILM and RPE was extracted at 50th percentile of the ILM and RPE column labels, and the BM was extracted from the 95th percentile of the RPEDC column labels, respectively. This shows the ILM, RPE, and BM boundaries, as described in the section “Evaluation of Segmentation Models—Volumetric Error–based Validation.” BM = Bruch’s membrane; ILM = internal limiting membrane; RPE = retinal pigment epithelium; RPEDC = retinal pigment epithelium drusen complex.

Figure 3 presents a qualitative depiction of the output from the 4 models for 3 patients with varying RPEDC volumes. B-scans captured by Spectralis and Cirrus devices showcase approximately the same retinal locations for each patient. Notably, the illustration reveals that the retinal layers in Spectralis B-scans exhibit slightly higher contrast compared with Cirrus B-scans. Additionally, the figure highlights the overall robust performance of all 4 models across patients with diverse drusen volumes, a factor that could introduce considerable challenges to the algorithms.

### Evaluation of Segmentation Models—Volumetric Error–Based Validation

For both the NSR volume (i.e., the volume between the ILM and the RPE layer) and the RPEDC volume (i.e., the volume between the RPE and the BM layer), we compared the tissue volumes predicted by 4 different models from Spectralis and Cirrus OCT images against the ground truth tissue volumes from reading center grading of the Spectralis OCT images (Fig 3A–D). Table 8 summarizes the overall mean ± standard deviation (in mm^3^) for each model. The models tested with Cirrus data have slightly higher errors compared with the equivalent models tested using Spectralis data. Overall, all 4 different models performed relatively robustly in terms of producing low volumetric errors irrespective of the test data being generated from different devices.

**Table 5:** Performance Comparison for U-Net and DeepLab Models Evaluated in CycleGAN-Based Domain-Adapted Cirrus Images

AMDSC Groups	ILM (in Pixels)		RPE (in Pixels)	
	MSE	MAE	MSE	MAE
CyleGAN + U-Net (Cirrus)				
Group 1	4.35 ± 1.51	4.13 ± 1.10	4.57 ± 1.54	4.40 ± 1.47
Group 2	4.64 ± 2.6	4.31 ± 1.77	5.02 ± 1.66	4.89 ± 1.89
Group 3	4.58 ± 2.43	4.38 ± 1.83	5.66 ± 1.89	5.10 ± 1.51
CyleGAN + DeepLab (Cirrus)				
Group 1	3.91 ± 1.13	3.58 ± 0.77	3.89 ± 0.93	3.68 ± 1.01
Group 2	3.86 ± 0.87	3.53 ± 0.65	4.42 ± 0.80	4.04 ± 0.89
Group 3	3.89 ± 1.04	3.47 ± 0.79	4.87 ± 1.28	4.37 ± 1.16

AMDSC = Age-related Macular Degeneration Severity Score; ILM = internal limiting membrane; MAE = mean absolute error; MSE = mean squared error; RPE = retinal pigment epithelium.

Mean square error and MAE for ILM and RPE layers for 3 different groups according to AMDSCs; 1 pixel *∼* 3.9 μm.

**Figure 4:**
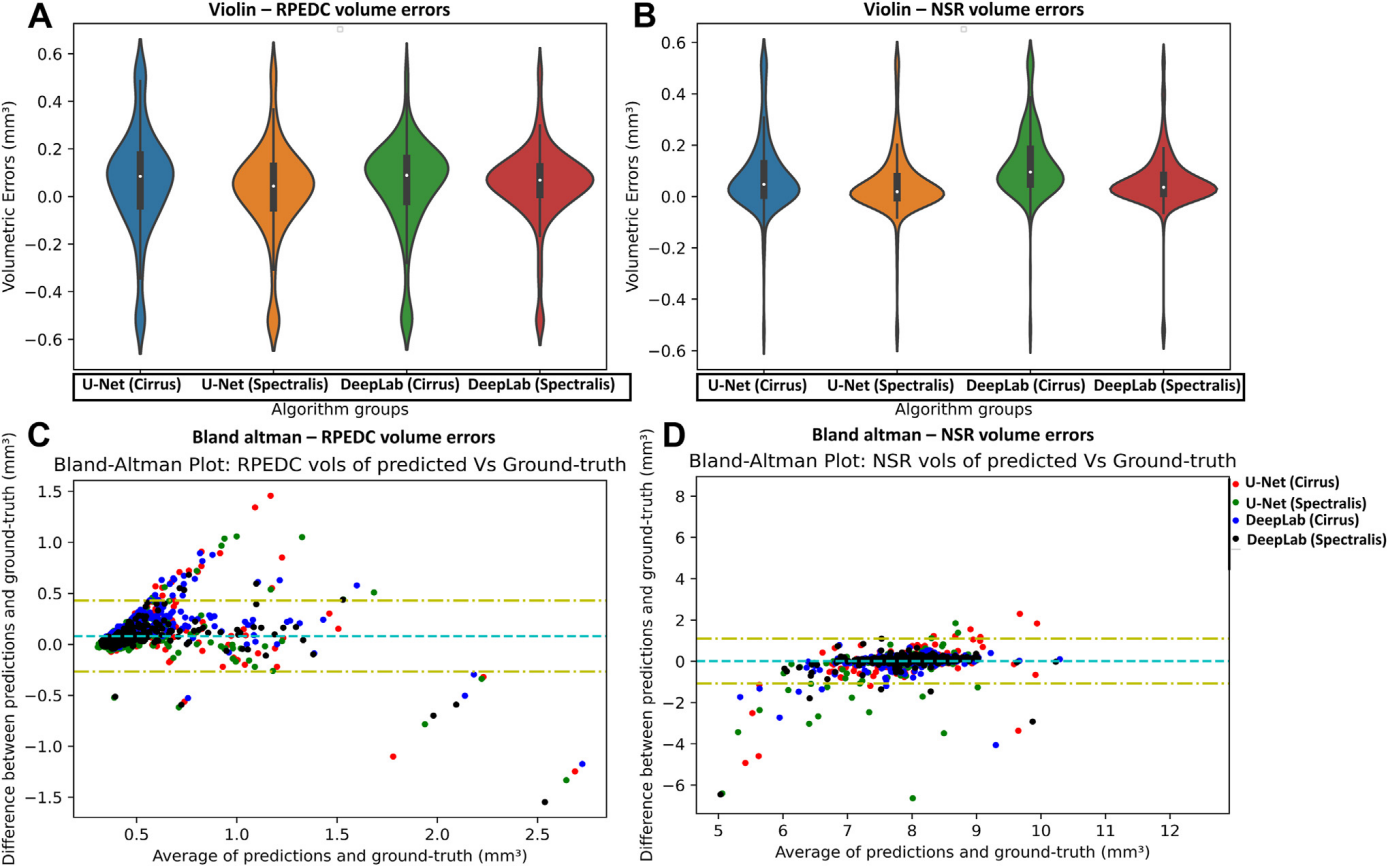
Predictions from 4 different models were compared against the reading center provided retinal volumes from the Spectralis images. U-Net (Cirrus), U-Net (Spectralis), DeepLabV3 (Cirrus), and DeepLabV3 (Spectralis). In (**A**) and (**C**) the RPEDC (from RPE to BM) errors denote the RPEDC volumetric errors, and in (**B**) and (**D**), the NSR (from ILM to RPE) errors denote the NSR volumetric errors. (**A**) and (**B**) show the violin plots of volumetric errors (mm^3^) between the 4 different prediction algorithms against the reading center–based ground truth. (**C**) and (**D**) show the Bland–Altman plots between the volumes generated using the 4 different prediction algorithms against the reading center–based ground truth. BM = Bruch’s membrane; ILM = internal limiting membrane; NSR = neurosensory retina; RPE = retinal pigment epithelium; RPEDC = retinal pigment epithelium drusen complex.

Violin plots of the errors and Bland–Altman plots are shown in Figure 4. For RPEDC volumes, the limits of agreement (LOA) were found to be between −0.28 mm^3^ and 0.43 mm^3^, and the mean error was 0.08 mm^3^. For NSR volumes, the LOA was found to be between −1.08 mm^3^ and 1.10 mm^3^, and the mean error was 0.01 mm^3^.

### RPEDC Thickness Map Analysis

The en face heat maps generated by U-Net (Cirrus) and DeepLabV3 (Cirrus) qualitatively resemble the manually created gold standard ground truth across a spectrum of AMDSCs and drusen volumes.

**Figure 5:**
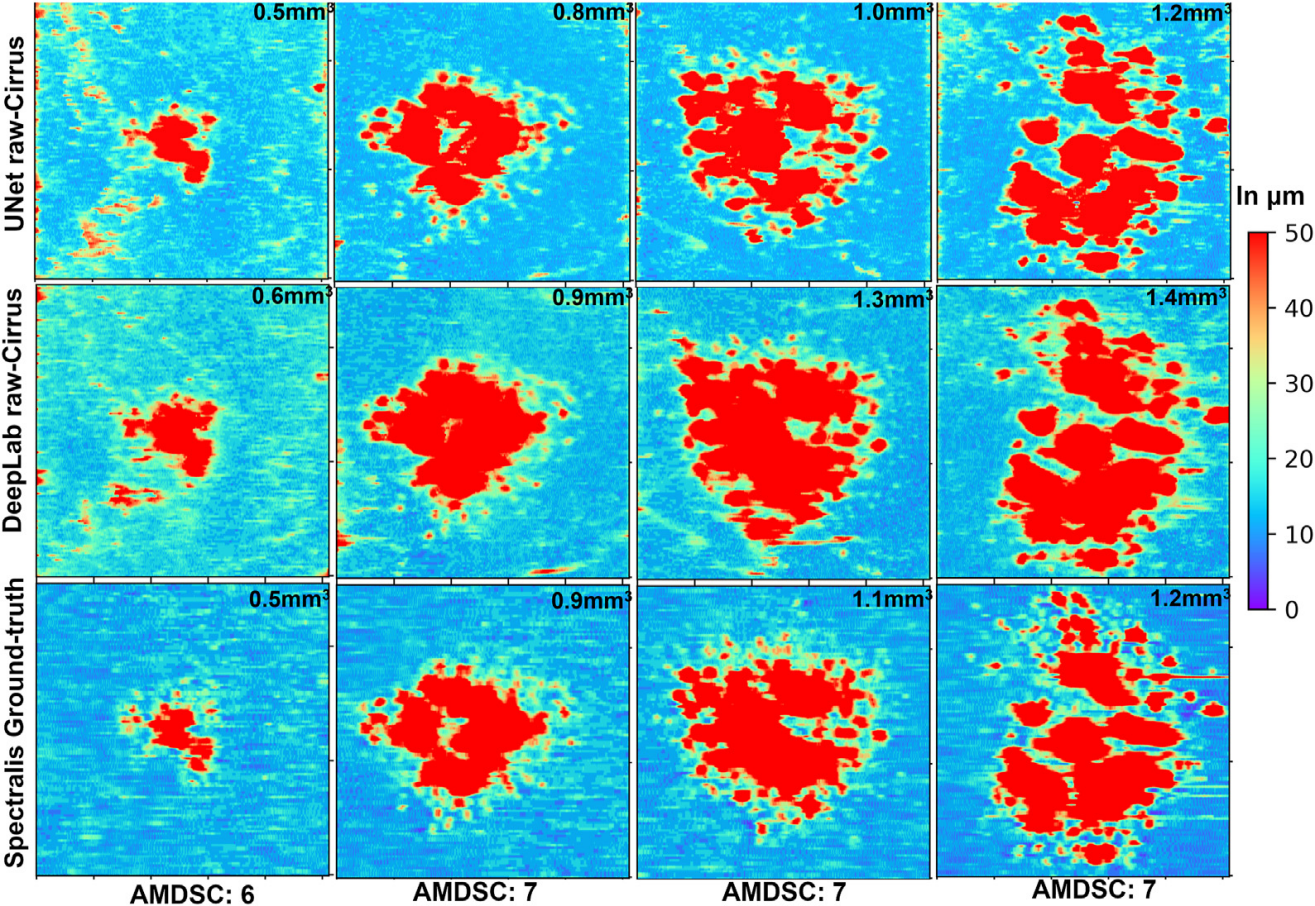
En face RPEDC thickness map comparison between 4 different cases with varying drusen volumes. Top row represents the 4 corresponding thickness maps created from the UNet (Cirrus) network predicted RPE and BM layers. Middle row represents the 4 corresponding thickness maps created from the DeepLabV3 (Cirrus) network predicted RPE and BM layers. Bottom row represents 4 thickness maps created from the Wisconsin Reading Center–based Spectralis ground truth of RPE and BM layers. The legend in the right most column represents absolute differences in μm or thicknesses between the RPE and BM layers, ranging from a minimum of 0 μm (deep blue) to a maximum of 50 μm (deep red). The AMDSCs at the bottom show the AMD severity grades provided by the reading center based on color photographs, and the numbers in the top right show the RPEDC volumes obtained from the model predictions from raw Cirrus OCTs and those provided by the reading center for the Spectralis OCTs. AMD = age-related macular degeneration; AMDSC = age-related Macular Degeneration Severity Score; BM = Bruch’s membrane; RPE = retinal pigment epithelium; RPEDC = retinal pigment epithelium drusen complex.

In Figure 5, we show an example of an eye with drusen with the corresponding en face heat maps illustrating the thicknesses of the RPE to BM layer for predictions generated by the U-Net (Cirrus) network (top row) and the DeepLabV3 (Cirrus) network (middle row). These predictions are juxtaposed against the ground truth provided by the Wisconsin Reading Center, utilizing Spectralis data (bottom row).

Table 4 presents the volumetric (since the RPEDC volume is a 3D volume) Dice similarity coefficients, assessing the agreement between RPEDC segmented regions predicted by 4 different algorithms and the corresponding ground truth regions. The groupings are based on AMDSCs as previously defined at the end of the section “Automatic SD-OCT Metrics and Validation.” The comparison of Dice similarity coefficients is conducted volumetrically. In general, both U-Net and Spectralis exhibit robust performance regardless of the test domain data, as indicated by the consistently fair Dice similarity coefficients reported in this table.

**Table 6:** Volumetric Dice Similarity Coefficients for RPEDC Region Segmentations for CycleGAN-Based Domain-Adapted Cirrus Images

AMD Groups	CyleGAN + U-Net (Cirrus): Annotator 1 and 2 (Aggregated)	CyleGAN + DeepLab (Cirrus): Annotator 1 and 2 (Aggregated)
Group 1	0.53 ± 0.13	0.73 ± 0.09
Group 2	0.57 ± 0.08	0.73 ± 0.05
Group 3	0.63 ± 0.08	0.75 ± 0.05

AMD = age-related macular degeneration; RPEDC = retinal pigment epithelium drusen complex.

A discernible decline in performance is noted as we transition to Group 3, encompassing patients with AMDSCs of 6, 7, and 8. This group is defined by having the largest drusen and largest drusen volumes with the most pronounced elevations of the RPE above the BM, thereby posing significant challenges to the algorithms.

### Comparison with CycleGAN-Based Domain Adaptation

The results presented in Table 5, Table 6 highlight the performance of U-Net and DeepLab models on CycleGAN-based domain-adapted Cirrus images (adapted to Spectralis). Across AMDSC groups, the DeepLab model consistently outperforms U-Net, when segmenting domain-adapted Cirrus images, showing lower MSE and MAE values for both the ILM and RPE layers. For instance, in Group 1, the DeepLab model achieves an MSE of 3.91 ± 1.13 for the ILM layer compared with U-Net's 4.35 ± 1.51 and a similar trend is observed across other groups. Furthermore, the Dice similarity coefficients in Table 6 indicate that DeepLab also provides higher accuracy in segmenting the RPEDC region, with scores reaching 0.75 ± 0.05 in Group 3, compared with U-Net’s 0.63 ± 0.08.

**Table 7:** Model Predicted MAE Comparison Showing *P* Values from a Paired 2-Tailed *t* Test

	Cirrus vs. Spectralis	
	ILM	RPE
UNet	0.11 ± 0.24	0.04 ± 0.16
DeepLabV3	0.06 ± 0.14	0.03 ± 0.07

ILM = internal limiting membrane; MAE = mean absolute error; RPE = retinal pigment epithelium.

**Table 8:** Volumetric Errors of 4 Different Prediction Algorithms with Respect to the Wisconsin Reading Center Generated Ground Truth Based on Spectralis Images

Networks/Volumes	U-Net (Spectralis)	DeepLabV3 (Spectralis)	UNet (Cirrus)	DeepLabV3 (Cirrus)
NSR (in mm^3^)	0.24 ± 0.63	0.16 ± 0.40	0.26 ± 0.62	0.18 ± 0.31
RPEDC (in mm^3^)	0.09 ± 0.16	0.08 ± 0.13	0.12 ± 0.19	0.14 ± 0.15

NSR = neurosensory retina; RPEDC = retinal pigment epithelium drusen complex.

Mean ± standard deviation (in mm^3^).

However, both networks demonstrated lower performance on CycleGAN-based domain-adapted images compared with raw Cirrus images, as the adaptation did not substantially improve segmentation accuracy. This decrease in accuracy may stem from limited control over the generated domain-adapted features, which may not sufficiently align with the segmentation task necessary for accurate layer segmentations by the backend segmentation algorithms. This result suggests that while CycleGAN can mitigate interdevice variability, further innovation may be necessary to optimize its compatibility with specific segmentation models like U-Net and DeepLab.

## Discussion

This work investigated the adaptability and generalization capabilities of deep neural networks, which are characterized by their extensive set of learnable parameters. Specifically, we evaluated whether these networks, widely employed for segmentation tasks, can transcend the inherent characteristics of imaging devices and effectively learn features that are independent of the training device’s source. These models, trained on a large dataset consisting of 25 728 B-scans, demonstrated robust performance when faced with previously unseen test domain data acquired from a different OCT device. The results indicate the potential significance of domain-independent task-specific neural networks for accurately segmenting retinal layers. Such networks may offer an advantage by alleviating the complexities and costs and efforts associated with domain adaptation.

Our demonstration reveals that both the U-Net and DeepLabV3 segmentation networks yield commendable segmentation results on test domain data obtained from a different device compared with the training domain data. These segmentation networks exhibit the ability to learn features from the training dataset that generalize effectively to out-of-domain test data acquired from a separate device, given enough training data to learn from.

Figure 1 highlights significant differences in intensities between the 28 annotated volumes acquired from different devices. The intensity histogram for Spectralis reveals a higher number of pixels with both lower and higher intensities compared with Cirrus, with fewer pixels showing intermediate intensities. However, since the exact postprocessing techniques employed by Heidelberg and Cirrus are proprietary and not publicly disclosed, the precise reasons for the observed differences in images remain unclear.

Although Spectralis images are visibly of higher quality than Cirrus images due to frame averaging and tracking, it is unclear whether this makes them superior for machine learning models. In fact, some machine learning models have been shown to perform better when noise is added to images during training to prevent overfitting.^23^^,^^24^ The aim of this article is to demonstrate that our model does not rely on features specific to Spectralis images, which are visibly of higher quality. The fact that our model was trained on averaged and tracked images but still made reliable predictions on unaveraged and untracked images highlights the robustness of our algorithm.

The outer retinal layers, including the RPE and BM, are most affected by AMD pathology and are the most challenging to segment in the presence of AMD. These layers, beginning with the photoreceptors, are crucial for visual function, making them some of the most clinically significant retinal layers in AMD. Numerous studies have focused exclusively on segmenting the outer retinal layers using deep learning algorithms.^7^^,^^25^, ^26^, ^27^, ^28^ We focus on segmenting the ILM, RPE, and BM in our article.

From Table 2, it can be noted that, for Cirrus test data, both models performed comparably and worse than the Spectralis test data. The degradation in performance could be due to image qualitative differences between the OCT devices. However, Table 3 shows that both models performed successfully compared with the human variability observed on Cirrus images. Additionally presented in Table 3 is the observation that groups characterized by advanced AMD, notably Group 3, displayed the lowest repeatability in human annotations. This can be ascribed to the influence of advanced disease features on the structure of retinal layers. Notably, ILM layer segmentation demonstrated minimal errors for both models and the 2 OCT imaging devices (Table 2). The ILM layer appears to be less susceptible to structural changes induced by the disease, resulting in superior repeatability and performance in both human annotations (Table 3) and algorithmic assessments (Table 2). Table 8 illustrates that while RPE segmentation accuracy impacts both NSR and RPEDC measurements, the marginally larger absolute volumetric errors in NSR, as opposed to RPEDC, may be attributed to a broader volumetric distribution in NSR volumes. Furthermore, the models evaluated with Cirrus data exhibit slightly higher errors compared with their counterparts tested with Spectralis data. This aligns with the distance-based metrics discussed in the section “Evaluation of Segmentation Models—Distance Error–based Validation” (Table 2) and could be influenced by disparities in image quality as well as the absence of a dedicated training dataset for Cirrus.

Segmentation accuracy for retinal layer segmentation using OCTs has been reported to have errors in the range of 1 to 2 pixels from the ground truth in traditional machine learning algorithms as in Agarwal et al,^29^ graph-based methods as in Hussain et al,^30^ and deep learning methods as in Mukherjee et al.^7^ In a recently published longitudinal deep network for retinal layer segmentation from OCTs,^31^ the mean absolute surface distances were found to be equivalent to 0.65 (standard deviation 0.14) pixels for ILM and 1.27 (standard deviation 0.333) pixels for the photoreceptor outer segment–RPE junction. However, these results were achieved when the training and test data came from the same device.

For our algorithm, the MAE is within 1 pixel for the ILM layer and within 2 pixels for the challenging RPE layer, when the DeepLabV3 (Spectralis) model is trained and tested using Spectralis data. However, when the model is transferred to Cirrus data, performance of DeepLabV3 (Cirrus) decreases to within 2.5 pixels for the ILM layer and within 3 pixels for the RPE layer. This decrease is understandable due to the domain shift between the training and test datasets. Our findings demonstrate that performance decreases when the test data come from a different device than the training data, but this decrease is within the range of 1.5 pixels from the original performance where train and test domain images are derived from the same imaging device. The novelty of our work lies in demonstrating that, with an extensive training dataset, it is possible to train a network to learn generalizable features that produce performance within 1.5 pixels from the results obtained when the training and test data come from the same domain.

Moreover, the MAE in the Cirrus dataset for both ILM and RPE layer predictions ranges from 1.82 to 3.56 pixels relative to the ground truth, respectively. The intergrader variability for these layers was found to be between 0.15 and 4.44 pixels. Our model’s performance falls within a similar range as the intergrader variability.

Retinal pigment epithelium drusen complex volume is an established structural metric for evaluating drusen load in 3D SD-OCT imaging.^32^, ^33^, ^34^, ^35^ Across all AMD stages, manual grading by experts at the Wisconsin Reading Center estimated an RPEDC volume of 0.46 ± 0.26 mm³, while algorithmic predictions yielded a slightly higher estimate of 0.54 ± 0.26 mm³. A different study on choroidal hypertransmission defects^36^ reported a drusen load of 0.332 ± 0.405 mm³ for iAMD eyes during the final visit before hypertransmission defects appeared. Similarly, the Age-Related Eye Disease Study 2 study^32^ found OCT drusen volumes in iAMD eyes to be 0.08 ± 0.16 mm³. The ability to apply algorithms across different instruments would allow for a comparison of features regardless of acquisition type. Currently, this is limited because each instrument employs distinct segmentation algorithms to segment varying retinal boundaries, resulting in different volume measurements. For instance, the proprietary Zeiss Cirrus software calculates RPEDC volumes based on the distance from the altered RPE layer to the expected location of the normal RPE, whereas other studies measure RPEDC regions using other layers.^37^^,^^38^ Moreover, directly comparing these values for 3D drusen load is challenging due to differences in the retinal areas analyzed—our study assessed the full 6-mm ETDRS circle, while others referenced earlier evaluated 5-mm-diameter circles only. The MACUSTAR study^39^ also reported the highest RPEDC volumes in the iAMD group (0.04 ± 0.07 mm³) but only within the inner 3-mm-diameter ETDRS circle. The LOA between predictions and ground truth for RPEDC volumes in our dataset ranged from −0.28 mm³ to 0.43 mm³, with ground truth values spanning 0.31 to 3.31 mm³. For cases with RPEDC volumes exceeding 3 mm³, larger errors within the LOA may be acceptable, as larger distributions tend to produce greater variability. However, in some cases with smaller drusen, higher errors were observed due to coexisting confounding factors such as advanced geographic atrophy, advanced neovascularization, or both.

The Dice coefficient reported in Table 4 is calculated from the entire volume, encompassing the complete set of B-scans for a visit, rather than being derived from an en face projection. Given that the RPEDC volume is a 3D structure, we compare the 3D regions to determine the Dice coefficient, rather than calculating it individually for each B-scan. Since the Dice coefficient does not account for true negatives, it could be misleadingly impacted by B-scans with very small RPEDC areas. Therefore, to generate an accurate Dice coefficient, we compare the 3D RPEDC volumes between the predictions and the ground truth.

Quantitatively, our assessment focuses on the performance metrics of 2 widely used models, UNet (Cirrus) and DeepLabV3 (Cirrus). These models exhibit successful performance across a spectrum of metrics, including moderate LOAs for NSR and RPEDC volumes and low MSEs and MAEs for ILM and RPE layer segmentations. Additionally, favorable Dice coefficients for RPEDC region segmentations affirm the robustness of these models. Furthermore, the translation of quantitative success to qualitative insights is evident in visually consistent segmentation results and RPEDC thickness-based heat maps. These visual representations provide an additional layer of confidence in the model’s ability to perform effectively across a different imaging device and a large dataset.

We acknowledge that a domain adaptation method could be an approach to increase the test performance of an algorithm developed on a different data domain. However, the goal of our article is to demonstrate that, with an extensive training dataset, deep learning models can learn generalizable features capable of producing clinically relevant results without the additional burden of training a domain adaptation network. Additionally, it is unclear how unpaired domain adaptation networks like CycleGAN alter extremely challenging biomarkers, such as small drusen, reticular pseudodrusen, or hyperreflective foci, which can be confounding even to expert human graders. Recent advancements in foundational models, both within^40^ and beyond the field of retinal imaging, including segment-anything models,^41^^,^^42^ are other viable approaches to solve the task of segmentation and have the potential to yield good results.

We acknowledge the substantial challenges posed by disease features, such as drusen, in achieving robust performance. The impact of these features on RPE segmentation is notable, with dynamic changes in reflectivity or thickness of the RPE layer. Despite these challenges, our findings underline the resilience of these deep neural networks. The accurate and robust performance across the dataset, even in the presence of challenging disease features, emphasizes their ability to learn intricate and generalizable features when provided with ample ground truth data. From Table 1, it can be noted that the DeepLabV3 model performed slightly better on the Spectralis test data. This could be due to larger fields-of-view enabled by a-trous filters (i.e., larger receptive fields of filters) that could aid in providing additional global context to robustly segment layers, for example, in the presence of drusen-induced fluctuations.

Retinal layer segmentation in domains lacking ground truth by transferring previously trained artificial intelligence models holds significant clinical relevance, particularly in the early detection, diagnosis, and monitoring of retinal diseases such as AMD. By accurately delineating the individual layers of the retina, algorithm-driven segmentation allows clinicians to quantify drusen volumes, which are a key biomarker in AMD. Moreover, algorithmic segmentation can standardize measurements across large datasets, improving the consistency and reliability of assessments. This will become particularly valuable as clinical trials focused on iAMD are on the horizon, which will likely focus on patients with a threshold amount of drusen volume and monitor drusen volume longitudinally. Ultimately, as treatments are developed, the integration of machine learning in retinal layer segmentation has the potential to lead to improved patient outcomes through more accurate, timely, and personalized care.

This study performed retinal layer segmentation using Spectralis and Cirrus data, employing 2 advanced segmentation networks. The segmentation process involved dissecting B-scans through the application of segmentation networks. These networks were trained only using Spectralis B-scans, along with corresponding ground truth annotations generated by the reading center. The segmentation specifically focuses on delineating the ILM, RPE layers, and the area spanning from the RPE to BM (RPEDC). One of the important aspects of our findings is the demonstration that training segmentation-based models with data originating from a distinct device domain can yield clinically valuable results, when applied to a different device domain. This is an important finding as it reinforces the potential of these segmentation networks to generalize effectively across different devices, even when trained on data from an entirely separate device domain. The implications of this extend beyond theoretical considerations, as our study substantiates the practical utility of such models in producing accurate and clinically relevant segmentation results.

## Data Availability

Data underlying the results presented in this paper are not publicly available at this time but may be obtained from the authors upon reasonable request.
